# Elbow Posterolateral Rotatory Instability due to Cubitus Varus and Overuse

**DOI:** 10.1155/2018/1491540

**Published:** 2018-08-05

**Authors:** Juan Martín Patiño, Alejandro Rullan Corna, Alejandro Michelini, Ignacio Abdon, Alejandro José Ramos Vertiz

**Affiliations:** Hospital Militar Central, Buenos Aires, Argentina

## Abstract

A malunion as a complication of distal humerus fractures has been frequently linked with aesthetic problems but less frequently with posterolateral rotatory instability. We report 2 cases of childhood posttraumatic cubitus varus with subsequent posterolateral rotatory instability and their treatment with a minimum of 2 years of follow-up. The etiology of the so-called posterolateral rotatory instability of the elbow is mostly traumatic, but iatrogenic causes have also been described such as the treatment of tennis elbow and less frequently and chronically due to overuse and overload because of distal humerus malunion.

## 1. Introduction

A malunion as a complication of distal humerus fractures has not only been frequently linked with aesthetic problems but also with instability and dislocation of the ulnar nerve [1], ulnar nerve neuritis [2], medial triceps dislocations [3], recurrence of distal humerus fractures or lateral condyle [4], osteoarthritis [5], and posterior dislocation of the radial head but less frequently with posterolateral rotatory instability [6–11].

The objective of this presentation is the report of 2 cases of childhood posttraumatic cubitus varus with subsequent posterolateral rotatory instability and their treatment with a minimum of 2 years of follow-up.

## 2. Case Report

### 2.1. Case 1

A 29-year-old female patient consults for pain and paresthesias in the 4th and 5th fingers with 2 years of evolution with several minor traumas in the past year. The patient had a history of supracondylar elbow fracture at the age of 5, treated nonsurgically. No symptoms were presented until she started with higher activity and physical demand such as bar exercises and push-ups. With the beginning of these symptoms, she was initially treated at another hospital for epicondylitis with physiotherapy, rest, and 2 corticoid injections without remission of symptoms.

Physical examination showed pain, an evident varus deformity, chair sign positive, and clear pivot shift. In anteroposterior radiograph, varus of 20 degrees and paresthesias in the ulnar nerve territory were observed. Electromyogram reported signs consistent with ulnar nerve entrapment. Her range of motion in flexion extension and supination was complete (grades: 0-145 flexion-extension, 50-50 pronosupination). The MEPI (Mayo Elbow Performance Index) was 60.

#### 2.1.1. Treatment

Valgus osteotomy was performed in the distal humerus through a lateral wedge and ligament reconstruction with tendon graft of the autologus palmaris longus, by tunneling the distal humerus and ulna crest. In rehabilitation, the range of motion was controlled with an articulated splint.

#### 2.1.2. Complications

Postoperative complications were delayed union and radial neuropraxia with spontaneous remission after 3 months.

#### 2.1.3. Result

**Figure 1:**
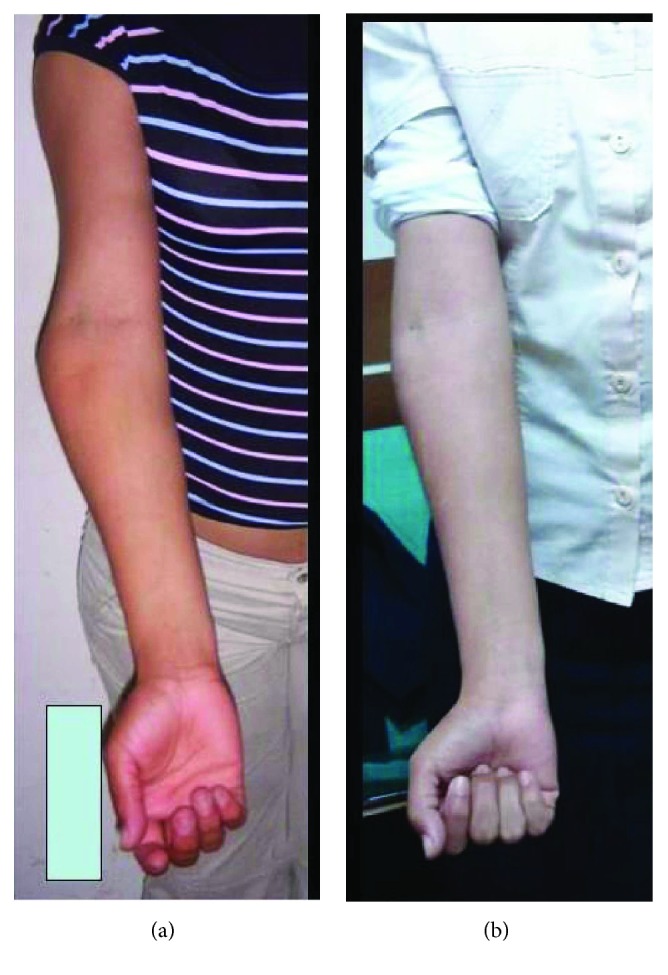
Case 1. Clinical appearance: (a) preoperative and (b) postoperative.

**Figure 2:**
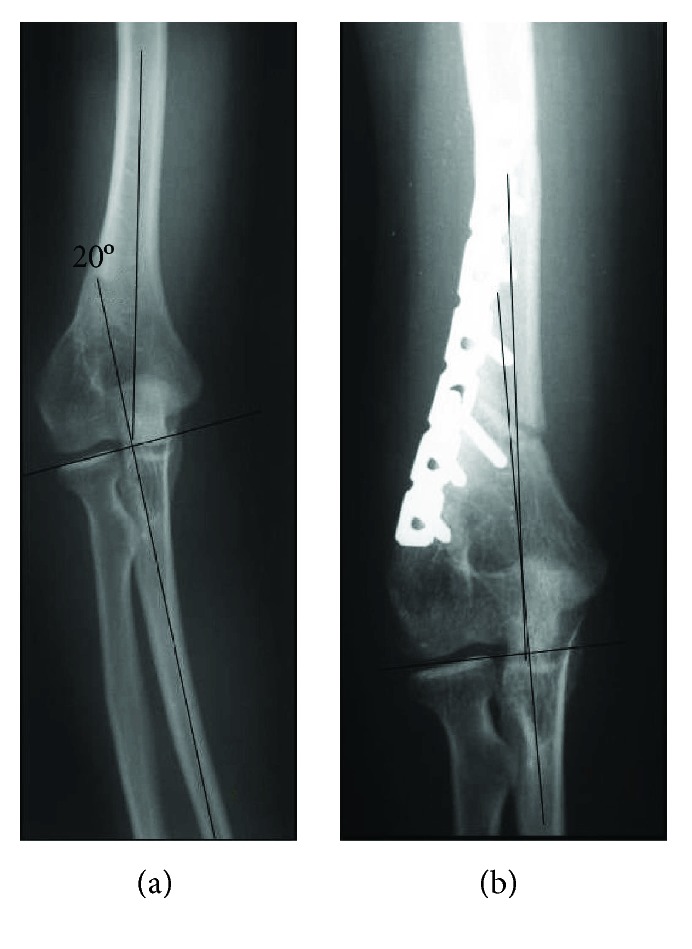
Case 1. (a) Preoperative AP radiograph. (b) Postoperative AP radiograph.

The osteotomy did not lead to valgus but to a correction of 5 degrees of the varus (previously 20 degrees). In the evaluation, after 4 years of follow-up, partial clinical deformity correction, remission of symptoms of ulnar nerve irritation, and complete range of motion were achieved. However, the patient cannot perform some exercises with high force demand or more than 2 hours of continuous activity. The MEPI was 80 and DASH (Disabilities of the Arm, Shoulder, and Hand) was 13.33 (Figures 1 and 2).

### 2.2. Case 2

A 19-year-old female patient consults for lateral elbow pain and functional limitation with 3 months of evolution. She mentions a history of elbow fracture when she was 4 years old (apparently lateral condyle) treated nonsurgically. She did not have previous symptoms. These appeared with increased elbow overload because of physical activity when entering the Military Academy. Physical exam showed pain and sign of instability such as positive pivot shift, which had to be confirmed under fluoroscopy; clinical attitude in the elbow varus was less evident than in the first case. In the anteroposterior radiograph, 10-degree varus was observed. MRI informed signs of chondral injuries in the radial head and the lateral collateral ligament, too. The MEPI was 65.

#### 2.2.1. Treatment

A lateral ligament reconstruction with autologous graft of palmaris longus was performed with similar technique of the first case and also capsular plication. The repair was protected with a transarticular nail for 3 weeks. Then, she began with progressive rehabilitation.

#### 2.2.2. Result

**Figure 3:**
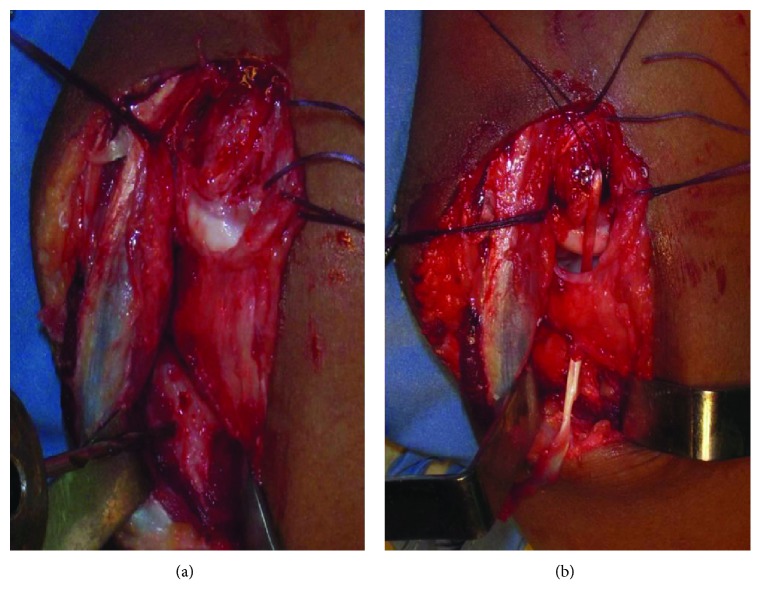
Case 2. (a, b) Collateral ligament reconstruction.

**Figure 4:**
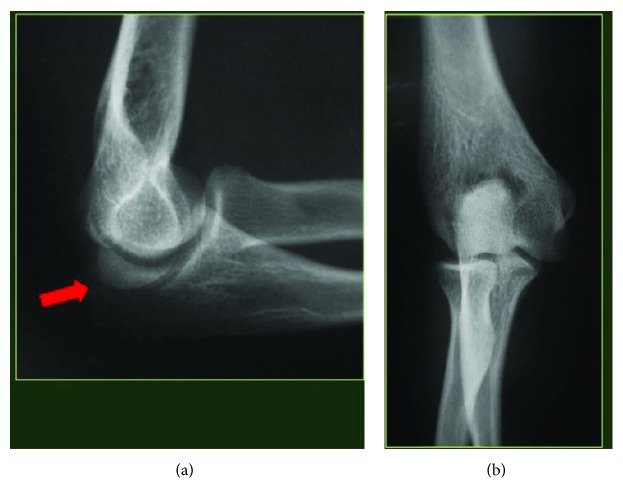
Case 2. (a, b) L and AP preoperative X-rays.

**Figure 5:**
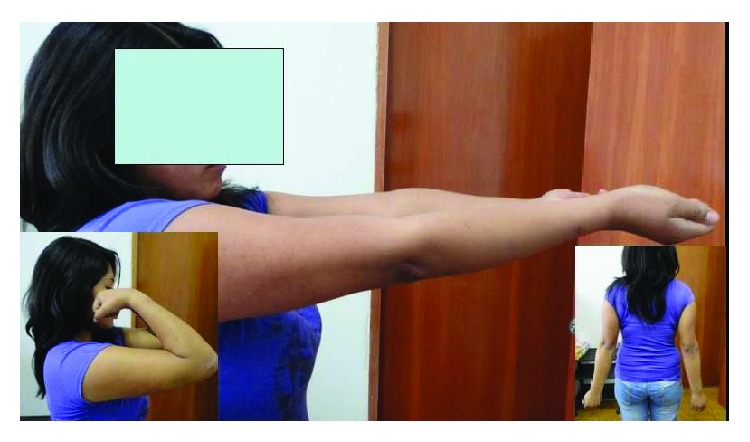
Case 2. Final elbow range of motion.

A stable elbow, full flexion and extension range, and full pronosupination were achieved. After 2 years of follow-up, the MEPI is 100 and DASH 0. She was capable of performing all daily life activities (Figures 3–5).

## 3. Discussion

In the cases presented, improvement was achieved in the stability of the elbow, though with better evolution in case 2 in which the deformity was less marked and the osteotomy was not necessary since the patient was active without limitations.

Bibliography on this subject is scarce. Elbow varus sequelae due to distal humerus fractures and its treatment is mainly related to aesthetic problems and functional issues [12, 13]. We have identified few experiences/publications and these mainly limited to case presentations.

Mondoloni et al. in 1995 [7] published 2 cases of instability in adults with antecedent of elbow supracondylar fractures in which stability and good motion with pain remission were achieved.

Abe et al. in 1997 [6] reported one 16-year-old patient with posterolateral instability, cubitus varus, and a history of distal humerus supracondylar fracture at the age of 5, which had shown no complications until he started playing volleyball with more elbow demand. He underwent ligament reconstruction and external osteotomy and osteosynthesis. He started playing again 5 months later without pain. After 10 months, he showed no instability, same preoperation motion, but with 23 degrees of valgus overcorrection.

O'Driscoll et al. [8] in a multicentric study studied 24 patients with 25 elbows with posterolateral instability and post fracture cubitus varus in 22 cases and congenital in 3. The instability appeared 2 or 3 decades after the deformity. Besides, all patients presented pain and the varus range was between 15 and 35 degrees. 22 cases underwent surgery: ligament reconstruction and osteotomy in 7 cases, ligament reconstruction as a unique procedure in 10, only osteotomy in 4, and arthroplasty in 1. In 3 cases, the triceps was electrically stimulated in surgery with resistance to extension, and elbow dislocation was observed.

Good or excellent results were achieved in 19 cases; in 3 cases, there was persistent instability. It is concluded that in the cubitus varus, the mechanical axis, the olecranon, and the triceps axis are displayed medially. This causes repetitive cubitus external rotation that can stretch the complex lateral ligament and cause posterolateral instability. The secondary cubitus varus malunion does not always imply benign lesion; it may present long-term symptoms, but these can be solved with surgery.

In this publication [8], a physiopathological explanation is proposed where the cubitus varus due to the malunion of the distal humerus causes 2 biomechanical alterations that alter the complex lateral ligament. Firstly, with the varus, the mechanical axis between the shoulder and the wrist moves medially. The repeated varus torque increases the lateral ligament stress especially with axial force on the limb (as it occurs when standing after having been seated on a chair). Secondly, the varus also displaces the medial triceps force vector leading the cubitus to move in this direction and to rotate externally to 90 degrees of flexion. Both are complementary reasons to the previously described explanations for instability [14]. This explanation was confirmed in a cadaveric study in which valgus osteotomies were practiced in 11 elbows, and when exposed to force, an increased lateral tension with larger varus was observed [15].

Ligament reconstruction as a unique procedure is suggested in varus below 15 degrees in the elderly or low force demand (not athletes) patients because without osteotomy, stress on the reconstruction increases.

The valgus osteotomy helps in stabilizing ligamentous laxity. The osteotomy as a unique procedure is feasible in cases with low instability and low demand to the elbow. In larger deformities, it has been suggested to combine osteotomy with the ligament reconstruction, as separately they would be related to a higher failure rate as well as to osteotomies which do not restore the valgus.

Elbow lateral pain is a reason of frequent consultation. Due to the fact that pain often appears as a symptom of instability, we consider that this pathology should be suspected and investigated, especially in cases wherein pain is associated with physical demand and traumatic events in childhood with sequels in the elbow.
